# Changes of Blood Pressure and Hemodynamic Parameters after Oral Magnesium Supplementation in Patients with Essential Hypertension—An Intervention Study

**DOI:** 10.3390/nu10050581

**Published:** 2018-05-08

**Authors:** Nikolina Banjanin, Goran Belojevic

**Affiliations:** Institute of Hygiene and Medical Ecology, Faculty of Medicine, University of Belgrade, 11000 Belgrade, Serbia; goran.belojevic@med.bg.ac.rs

**Keywords:** magnesium, dietary supplements, hypertension, systemic vascular resistance, cardiac output

## Abstract

The objective of this study was to examine the changes of blood pressure and hemodynamic parameters after oral magnesium supplementation in patients with essential hypertension. The single-arm non-blinded intervention study comprised 48 patients (19 men; 29 women) whose antihypertensive therapy was not changed for at least one month. The participants were asked to consume (daily at home) 300 mg of oral magnesium-oxide supplementation product for one month and to have their blood pressure and hemodynamic parameters (thoracic fluid content, stroke volume, stroke index, cardiac output, cardiac index, acceleration index, left cardiac work index and systemic vascular resistance index, heart rate) measured in the hospital before and after the intervention. Measurements were performed with impedance cardiography. After magnesium supplementation, systolic and diastolic pressures were significantly decreased (mean ± standard deviation (SD)/mmHg/from 139.7 ± 15.0 to 130.8 ± 13.4 and from 88.0 ± 10.4 to 82.2 ± 9.0, respectively; both *p* < 0.001). The two significant hemodynamic changes were the decrease of systemic vascular resistance index (dyn s m^2^/cm^5^) and left cardiac work index (kg m/m²)/mean ± SD from 2319.3 ± 753.3 to 2083.0 ± 526.9 and from 4.8 ± 1.4 to 4.4 ± 0.9, respectively; both *p* < 0.05). The observed hemodynamic changes may explain lowering blood pressure after magnesium supplementation.

## 1. Introduction

Magnesium is an important cation for the activity of many enzymes related to energy metabolism, e.g., serine racemase [1], ATP diphosphohydrolase [2] or myosin ATPase [3]. In addition, elevated levels of serum magnesium may lead to smooth muscle relaxation and vasodilatation due to antagonistic action on calcium receptors and channels [4]. On the other hand, the reduction of extracellular magnesium causes vasospasm, particularly important pathogenic factor for sudden death in ischemic heart disease [5] and for glaucoma and diabetic retinopathy [6]. Elevated extracellular magnesium leads to decrease of blood pressure through stimulated production of prostacyclin in endothelial cells and vascular smooth muscle cells [7] and the inhibition of norepinephrine release from nerve endings [8].

An intervention study of a 6-month supplementation with magnesium aspartate hydrochloride among patients on a diuretic therapy for hypertension showed a significant reduction of both systolic and diastolic blood pressure [9]. On the other hand, a double-blind randomized study reported no effect of a three-month magnesium supplementation on blood pressure when compared with placebo treatment [10]. A recent meta-analysis of randomized double-blind placebo controlled trials showed that a daily supplementation with a dose of 300 mg magnesium of at least one month duration significantly reduced blood pressure [11]. The discrepancies in the presented studies may be explained by the differences regarding the study designs, study protocols, ways, form or duration of supplement application, age and race of participants, patients’ compliance, the instruments of measurement and the inclusion and exclusion criteria.

Due to conflicting results there is a lack of definitive conclusion and consensus on the effectiveness of magnesium in the treatment of hypertension. Besides blood pressure there is a need for further investigation of the effects of magnesium on other hemodynamic parameters that may explain the Mg-hypertension relationship. The aim of this intervention study was to examine the changes of blood pressure and hemodynamic parameters after oral magnesium supplementation in patients with essential hypertension. We hypothesized that magnesium supplementation would lead to lowering blood pressure and other compatible hemodynamic changes.

## 2. Materials and Methods

This study was performed in the collaboration between the Institute of Hygiene with Medical Ecology, Faculty of Medicine, and the Multidisciplinary Center for Polyclinic Diagnostics, Assessment and Treatment of Blood Pressure Disorders, Clinic for Cardiology, Clinical Center of Serbia. The study has been registered by The Iranian Registry of Clinical Trials (www.irct.ir; Registration number: IRCT2017081535716N1). All subjects signed their informed consent for inclusion before they participated in the study. The study was conducted in accordance with the declaration of Helsinki, and the protocol was approved by the Ethics Committee of the Clinical Center of Serbia, Belgrade (code 1322/1) and by the Ethics Committee of the Faculty of Medicine, University of Belgrade, Serbia (code 29/VII-16).

The investigation was designed as a single-arm non-blinded intervention study due to an expected limited pool of patients that would fulfill all inclusion and exclusion criteria [12]. The recruitment of patients was performed from September to November 2014 and comprised all patients aged 24–65 years with essential hypertension who came for a regular blood pressure check-up. Among them, only patients whose antihypertensive therapy was not changed for at least one month were eligible for the intervention study. The patients were not allowed to change their blood pressure medication during the trial. The exclusion criteria for participation in the study were: renal diseases, gastrointestinal diseases, diabetes mellitus, diseases of adrenal, thyroid and parathyroid glands, angina pectoris, myocardial infarction, coronary revascularization, congestive heart failure, aortic coarctation, food and drug allergy, pregnancy and lactation, transitory ischemic attack and oral intake of magnesium supplements in the previous month.

The minimum sample size of 43 patients was calculated based on previously reported clinically significant reduction of mean values of systolic and diastolic pressure of 3 mmHg and 2 mmHg, respectively, and effect size of 0.44 after magnesium supplementation [13]. The calculation was performed online using the input values of a power of 80% and a two sided level of significance of 5% [14].

The eligible participants (*n* = 68, 27 men and 41 women) were asked to consume 300 mg of oral magnesium-oxide supplementation product for one month and to have their blood pressure and hemodynamic parameters measured in hospital settings at baseline and at the end of the intervention. The participants were given a dietary supplement (“Magnezijum 300 Direkt”, Hermes Arzneimittel GmbH, Wolfratshausen, Germany) which is approved by the Ministry of Health of the Republic of Serbia (Number: 1708/2011). All participants were informed how to use this product, were warned not to exceed the daily recommended dose, and were advised not to change their dietary habits. The participants did not report adverse effects that might be related to magnesium supplementation. They were not informed about the expected changes of blood pressure and hemodynamic parameters after magnesium supplementation.

At baseline, the participants underwent a detailed medical examination and completed a questionnaire that included personal data (age, gender), duration of hypertension (years), hypertension in the family (coded as: 1-any family member diagnosed with hypertension; 0-none), physical activity of at least 30 min most days per week (coded as: 1-Yes; 0-No) and smoking habits (coded as: 0-non-smoker;1-current smoker; 2-ex-smoker). Data on antihypertensive treatment were obtained from patient’s medical records. Antihypertensive treatment included: ACE inhibitors (23 patients; 48%); calcium channel blockers (24;50); thiazide diuretics (24;50); beta blockers (35;73) and angiotensin II receptor blockers (10;21). At follow-up after one month, all participants were invited for medical examination. Despite being called several times, 20 participants did not return for check-up (8 men and 12 women), but they did not report adverse effects of magnesium supplementation as the reason. The statistical analysis revealed that the non-responders were significantly younger than the responders (mean age of the non-responders 37.75 ± 12.45 years) with non-significantly shorter duration of hypertension (mean duration of hypertension among the non-responders 5.39 ± 5.19 years). The final sample comprised 48 persons, 19 men and 29 women, aged 47.40 ± 11.30 years, range 24 to 65 years. This sample size was considered sufficient according to afore mentioned calculation.

Average daily magnesium intake from food and drinks was obtained using a 24-h recall questionnaire. The participants were very carefully asked about food and drinks they consumed during 24 h prior to the interview. The questions referred to food types and amounts, time and place of consumption, how the food was prepared (fried, fresh, baked or cooked) and what was used to prepare the food (salt, oil, sugar etc.). A trained physician conducted the interview with the participants. Magnesium intake from food and drinks was calculated from all foodstuffs consumed the previous day using Serbian food tables [15]. Serum magnesium concentration was measured both at baseline and follow up using the photometric method [16]. Blood samples were taken at the central laboratory of the Clinical Center of Serbia between 8 and 9 a.m., after a morning fast. The laboratory is an accredited institution with a strict quality control standards for all laboratory analyses.

Participants had their weight and height measured by a trained physician. Body height was measured to the nearest 0.5 cm. Body weight was measured on a digital scale (TANITA Inner Scan Body Composition Monitor BC-543) to the nearest 0.1 kg. Participants were in light clothes and barefoot. Body mass index (BMI) was calculated as body weight (in kilograms) divided by squared body height (in meters).

Both at baseline and at follow-up participants had their systolic, diastolic and mean arterial blood pressure measured with impedance cardiography (ICG) by CardioScreen^®^ 2000 (Medis. Medizinische Messtechnik GmbH, Ilmenau, Germany). A cuff was placed on patient’s left upper arm.

We also used ICG for measurements of hemodynamic parameters at baseline and follow-up. ICG electrodes measure the change in thorax impedance during the flow of the alternating current through the thorax. The following parameters were measured: thoracic fluid content (the electrical conductivity of the chest, L/kOhm), acceleration index (the acceleration of blood in the ascending aorta and the aortic arch, L/100/s^2^) and heart rate (beats per minute). The following parameters were calculated: stroke volume (the amount of blood pumped by the left ventricle each beat, mL) and cardiac output (stroke volume multiplied by heart rate, L/min). With reference to body surface area the following parameters were calculated: stroke index (stroke volume related to the body surface, mL/m^2^), cardiac index (cardiac output related to the body surface, L/(min m^2^), systemic vascular resistance index (the resistance to the blood flow through the arterial system the heart works against, dyn s m^2^/cm^5^) and left cardiac work index (an expression for the work of the left ventricle, kg m/m²). DuBois formula was used to calculate the body surface area [17].

Descriptive statistic was presented as mean values and standard deviation for numeric variables, or as percent’s (relative numbers) for categorical variables. All investigated parameters were tested by Kolmogorov-Smirnov test and almost all observed distributions corresponded to the normal distribution. The differences between men and women were tested with Student’s t-test for parametric variables and with Mann-Whitney *U*-test and Chi-square test for non-parametric data. The differences between baseline and follow-up were tested with paired-samples *t*-test. Probability level of less than 0.05 was accepted as significant. Statistical analyses were performed using SPSS 15.0 for Windows software (SPSS Inc., Chicago, IL, USA, 1989–2006).

## 3. Results

Men and women were of similar age, and had similar body mass index, blood pressure levels, and shared smoking habits, engagement in physical activity and magnesium intake from food and water and serum magnesium concentrations. Men and women had similar duration of hypertension, and had similar family history of hypertension. These results support further analysis regardless of gender (Table 1).

There was no significant difference between serum magnesium concentrations at baseline and at follow-up (mean ± SD = 0.858 ± 0.054 and 0.847 ± 0.070 mmol/L, respectively; *p* = 0.432). The range of serum magnesium concentrations at baseline and at follow-up was 0.74–0.94 and 0.69–0.96 mmol/L, respectively.

Magnesium supplementation lead to significant decrease in systolic, diastolic and mean arterial blood pressure. Mean systolic pressure was decreased by 8.97 ± 2.01 mmHg; mean diastolic pressure was decreased by 5.87 ± 1.49 mmHg; mean arterial pressure was decreased by 8.06 ± 1.67mmHg (Table 2).

After one month of supplementation, thoracic fluid content, stroke index, cardiac index acceleration index and heart rate remained stable. On the other hand, systemic vascular resistance index and left cardiac work index were significantly decreased following a month of magnesium supplementation (Table 3). Mean change of left cardiac work index equaled 0.439 ± 0.19 kg m/m^2^, whereas mean change of systemic vascular resistance index was 236.29 ± 93.49 dyn s m^2^/cm^5^.

## 4. Discussion

We show a reduction of blood pressure after a one-month magnesium supplementation in hypertensive patients. This effect is compatible with a reduction in two hemodynamic parameters: systemic vascular resistance and left cardiac work.

Our results are in accordance with a meta-analysis of 22 trials which showed that the intake of magnesium supplements exceeding 370 mg daily has a bigger effectiveness in the reduction of systolic and diastolic blood pressures in hypertensive patients than the dose lower than 370 mg daily. The study found that the average reduction of systolic blood pressure was 3–4 mmHg and diastolic 2–3 mmHg after the consumption of magnesium supplements lasting from 3 to 24 weeks (average duration 11.3 weeks) [13]. Another review reported that oral magnesium supplements at a dosage from 10 to 15 mmol per day may reduce blood pressure in hypertensive patients on antihypertensive therapy [18].Additionally, our study points out the changes in hemodynamic parameters after magnesium supplementation that are compatible with lowering blood pressure. But, the results of the studies on the association between magnesium intake and blood pressure are not fully congruent. For example, a double-blind randomized study was conducted in patients with essential hypertension. When treatment with 40 mmol (972 mg) magnesium aspartate supplementation daily was compared with placebo treatment for three months no effect of magnesium supplementation on blood pressure was found [10]. However, this study had a small sample size of 13 patients. Another experimental study conducted in the US showed that magnesium supplementation for 16 weeks in dose of 14 mmol (336 mg) of magnesium had no blood pressure-lowering effect in normotensive women who reported low habitual intake of this mineral in comparison with placebo group [19].

An important magnesium function, the vasodilatation of peripheral arteries [20], may explain the lowering of systemic vascular resistance in our study. Magnesium may achieve its vasodilatatory effect through its impact on: intracellular calcium concentration [21], production of prostacyclin [7], and sensitivity to angiotensin II [22].

Serum magnesium concentration was not significantly changed after magnesium supplementation in our study. This finding is congruent with other intervention studies of a similar duration [23,24]. A significant raise of serum magnesium concentration after a seven-week magnesium supplementation may be expected only in participants with hypomagnesemia (concentrations lower than 0.70 mmol/L) [23] but in our sample the lowest serum magnesium concentration at baseline was 0.74 mmol/L. But, in longer intervention studies of a median duration of 12 weeks a significant elevation of serum magnesium concentrations after magnesium supplementation may be expected even in patients with baseline median serum magnesium concentrations of 0.785 mmol/L [25].

There are several limitations of this study. First, a single-arm non-blinded design was chosen because of a limited pool of patients. We consider that a placebo effect was alleviated by the fact that although the patients knew they were given magnesium supplementation they were not informed about the expected changes in blood pressure and hemodynamic parameters. Second, we did not control the possible effect of thiazide diuretics on magnesium excretion in some patients. Third, we did not check for magnesium concentration in a 24 h urine sample at baseline and at follow-up. Fourth, the intervention period of our study was limited to thirty days. In further investigations it would be useful to examine the effects of different duration (shorter and longer than in our study) of magnesium supplementation on blood pressure and hemodynamic parameters. Fifth, the study was conducted in persons with essential hypertension; the hemodynamic changes in persons with diabetes or kidney diseases would be of great importance for understanding the mechanisms of action of magnesium supplementation. The strength of this study is the use of ICG, which objectively measures both blood pressure and hemodynamic parameters that may explain blood pressure changes after magnesium supplementation.

## 5. Conclusions

In conclusion, oral magnesium supplementation probably had a positive effect on blood pressure reduction in patients with essential hypertension. The decrease in systemic vascular resistance and left cardiac work may explain the reduction of blood pressure after magnesium supplementation.

## Figures and Tables

**Table 1:** Baseline characteristics of the investigated population by gender.

Parameters	Men	Women	Total	*p* Value
Gender (%)	19 (39.6)	29 (60.4)	48 (100.0)	NS (*p* > 0.05) *
Age (years)	43.79 ± 11.11	49.76 ± 10.98	47.40 ± 11.30	NS **
Body mass index (kg/m^2^)	27.03 ± 2.48	25.94 ± 5.36	26.38 ± 4.42	NS **
Duration of hypertension (years)	9.50 ± 9.75	7.75 ± 6.99	8.44 ± 8.14	NS ***
Hypertension in the family (%)	15 (83.3)	25 (92.6)	40 (88.9)	NS *
Current smoker (%)	7 (36.8)	9 (31.0)	16 (33.3)	NS *
Regular physical activity (%)	10 (52.6)	18 (64.3)	28 (59.6)	NS *
Systolic pressure (mmHg)	140.74 ± 11.14	139.09 ± 17.26	139.74 ± 15.03	NS **
Diastolic pressure (mmHg)	87.37 ± 11.43	88.50 ± 9.83	88.05 ± 10.39	NS **
Mg intake from food (mg/day)	196.30 ± 114.74	158.73 ± 50.19	173.60 ± 82.99	NS ***
Mg intake from water (mg/day)	12.69 ± 6.44	15.55 ± 8.13	14.42 ± 7.56	NS ***
Total Mg intake (mg/day)	208.89 ± 115.66	174.28 ± 51.72	187.98 ± 83.72	NS ***
Serum magnesium concentration (mmol/L)	0.86 ± 0.05	0.85 ± 0.06	0.85 ± 0.05	NS **

* Chi-square test; ** Student’s *t*-test; *** Mann–Whitney *U*-test. NS (not significant)

**Table 2:** Changes of blood pressure after magnesium supplementation in patients with essential hypertension (*N* = 48).

Parameters	At Baseline	At Follow-Up	95% Confidence Interval of the Difference	*p* Value *
Systolic pressure (mmHg)	139.74 ± 15.03	130.77 ± 13.42	4.92–13.02	<0.001
Diastolic pressure (mmHg)	88.05 ± 10.39	82.19 ± 9.00	2.88–8.86	<0.001
Mean pressure (mmHg)	102.13 ± 12.07	94.07 ± 10.11	4.70–11.42	<0.001

* Paired-samples *t*-test.

**Table 3:** Changes of hemodynamic parameters after magnesium supplementation in patients with essential hypertension (*N* = 48).

Parameters	At Baseline	At Follow-Up	95% Confidence Interval of the Difference	*p* Value *
Thoracic fluid content (L/kOhm)	38.75 ± 8.95	36.57 ± 6.09	−0.67–5.04	0.131
Stroke volume (mL)	101.74 ± 27.90	99.26 ± 24.13	−10.18–15.13	0.686
Stroke index (mL/m^2^)	48.77 ± 11.31	48.44 ± 11.09	−5.62–6.28	0.905
Cardiac output (L/min)	6.93 ± 1.42	6.76 ± 1.29	−0.58–0.93	0.636
Cardiac index (L/(min m^2^))	3.61 ± 0.95	3.56 ± 0.64	−0.21–0.30	0.727
Acceleration index (L/100/s^2^)	105.96 ± 39.64	104.88 ± 40.27	−8.98–11.13	0.831
Left cardiac work index (kg m/m^2^)	4.83 ± 1.42	4.39 ± 0.91	0.05–0.82	0.026
Systemic vascular resistance index (dyn s m^2^/cm^5^)	2319.30 ± 753.31	2083.01 ± 526.95	47.87–424.71	0.015
Heart rate (beat/min)	71.56 ± 11.82	69.55 ± 9.06	−0.83–4.85	0.162

* Paired-samples *t*-test.
